# A Phosphorylcholine-Containing Glycolipid-like Antigen Present on the Surface of Infective Stage Larvae of *Ascaris* spp. Is a Major Antibody Target in Infected Pigs and Humans

**DOI:** 10.1371/journal.pntd.0005166

**Published:** 2016-12-01

**Authors:** Johnny Vlaminck, Dries Masure, Tao Wang, Peter Nejsum, Cornelis H. Hokke, Peter Geldhof

**Affiliations:** 1 Department of Virology, Parasitology and Immunology, Ghent University, Merelbeke, Belgium; 2 Department of Veterinary Disease Biology, Faculty of Health and Medical Sciences, University of Copenhagen, Denmark; 3 Department of Parasitology, Leiden University Medical Center, Leiden, The Netherlands; Centers for Disease Control and Prevention, UNITED STATES

## Abstract

**Background:**

The pig parasite *Ascaris suum* plays and important role in veterinary medicine and represents a suitable model for *A*. *lumbricoides*, which infects over 800 million people. In pigs, continued exposure to *Ascaris* induces immunity at the level of the gut, protecting the host against migrating larvae. The objective of this study was to identify and characterize parasite antigens targeted by this local immune response that may be crucial for parasite invasion and establishment and to evaluate their protective and diagnostic potential.

**Methodology/Principal Findings:**

Pigs were immunized by trickle infection for 30 weeks, challenged with 2,000 eggs at week 32 and euthanized two weeks after challenge. At necropsy, there was a 100% reduction in worms recovered from the intestine and a 97.2% reduction in liver white spots in comparison with challenged non-immune control animals. Antibodies purified from the intestinal mucus or from the supernatant of cultured antibody secreting cells from mesenteric lymph nodes of immune pigs were used to probe L3 extracts to identify antibody targets. This resulted in the recognition of a 12kDa antigen (As12) that is actively shed from infective *Ascaris* L3. As12 was characterized as a phosphorylcholine-containing glycolipid-like antigen that is highly resistant to different enzymatic and chemical treatments. Vaccinating pigs with an As12 fraction did not induce protective immunity to challenge infection. However, serological analysis using sera or plasma from experimentally infected pigs or naturally infected humans demonstrated that the As12 ELISA was able to detect long-term exposure to *Ascaris* with a high diagnostic sensitivity (98.4% and 92%, respectively) and specificity (95.5% and 90.0%) in pigs and humans, respectively.

**Conclusions/Significance:**

These findings show the presence of a highly stage specific, glycolipid-like component (As12) that is actively secreted by infectious *Ascaris* larvae and which acts as a major antibody target in infected humans and pigs.

## Author Summary

Roundworms infect millions of humans and pigs throughout the world. The pig roundworm *A*. *suum* is a good model for *A*. *lumbricoides* infection in humans due to similar host physiology and the close genetic relationship between the worms. The aim of this study was to identify and characterize early larval antigens that are targeted by antibodies at the level of the intestine in immune pigs and to evaluate their protective and diagnostic potential. In order to do so, we generated highly immune pigs by repeatedly infecting them with *A*. *suum* for a long time (32 weeks). After necropsy, locally harvested antibodies from the gut were used to screen larval extracts. Hereby one particular antigen, named As12, was detected. It was characterized as a molecule of glycolipid nature that is presented on, and actively secreted from, the surface of infective larvae. Pigs immunized with this antigen are not protected from subsequent challenge infection. Experimentally infected pigs or naturally infected humans do however mount a significant serological antibody response to the antigen. These findings shed light on a glycolipid-like antigen (As12) that is secreted by infectious *Ascaris* larvae and is targeted by the immune system of infected humans and pigs.

## Introduction

*Ascaris lumbricoides* is the most prevalent intestinal parasitic nematode of man, infecting approximately 819 million people worldwide in developing countries [1]. Due to the high degree of morphological and genetic similarity, it is still debated as to whether *A*. *lumbricoides* from humans is a different species than *A*. *suum* from pigs [2–4]. Moreover, recent studies have shown that pig *Ascaris* is a zoonosis [5–8]. Even though anthelmintic treatment remains highly effective against *A*. *lumbricoides*, there is increased concern about the development of anthelmintic resistance. In addition, the high risk of reinfections after treatment calls for the development of new, long-acting solutions like vaccination. In addition, the development of more rapid and sensitive diagnostic techniques that adequately reflect the level of *Ascaris* exposure in a population could greatly improve our knowledge on infection dynamics and prevalence. Consequently, it would thus allow for a more precise estimate of the impact of infection and a better evaluation of a given intervention.

Vaccination has proven to be the most efficient and cost-effective way of disease control [9]. Vaccination against ascariasis should in theory be feasible since pigs, repeatedly infected with *A*. *suum*, develop immunological responses at the level of the liver, lungs and intestine that stop migrating larvae from reaching adulthood. Furthermore, repeated exposure to the parasite induces an immunological response at the level of the intestine that is called the 'pre-hepatic barrier', eventually preventing newly acquired larvae from migrating to the liver [10, 11]. Recently, it has been shown that this immunity was associated with eosinophilia, mastocytosis and goblet cell hyperplasia in the caecum, the place where the infective stage 3 larvae (L3) penetrate the intestine and start their hepatopulmonary migration [12]. However, it is still unclear which parasite products induce these immune responses or what the targets of these responses are. An increased understanding on this matter could offer important information for the development of protection by vaccination against this parasite.

The need for improved methods to diagnose *Ascaris* infections in pigs and humans has recently been extensively discussed [13, 14]. It was suggested that diagnostic tools detecting eggs in the stool are not useful for accurate evaluation of the level of exposure in pig farms [15] or sensitive enough for the detection of infection in humans where prevalence was low [16]. Serological tools detecting exposure to *Ascaris* might be more sensitive than egg based diagnostics for measuring prevalence or intensity of exposure in a human community [17]. Until now, only a handful of studies report the evaluation of antibody-based tests for ascariasis [18–23]. Recently, Vlaminck et al., [15, 17] showed that an ELISA detecting antibodies to *Ascaris* haemoglobin in plasma or serum samples appears to reflect general exposure to *Ascaris* on a community or herd level in humans and pigs, respectively. However, more species-specific antigens from early larval stages might increase the sensitivity and specificity of serological assays or recognize infections at an earlier stage.

Hence, the main objective of this study was to use intestinal antibodies from pigs with a proven pre-hepatic barrier to identify immunogenic proteins of the infective stage larvae of *A*. *suum* and subsequently evaluate their protective and diagnostic potential.

## Methods

### Experimental animals

The piglets used in this study were female and castrated male Rattlerow Seghers hybrid pigs of the local stock of the animal facility (Ghent University). They were approximately 10 weeks old and weighed between 20 and 30 kg at the start of the trials. The pigs were raised indoors in a helminth free environment and had free access to a commercial feed and water.

### Parasite material

Adult female *A*. *suum* worms were collected from the intestines of naturally infected pigs from commercial farms that were being processed as part of the normal work at a local abattoir in Ghent, Belgium. Consent was acquired from abattoir management to collect the worms. Adult female *A*. *lumbricoides* worms were collected from the stool of individuals after treatment with anthelmintics in Jimma Town, Ethiopia [24].

*Ascaris* eggs were obtained by dissection of worm uteri and suspended in a 0.5% (w/v) potassium dichromate solution to a volume of 50 ml and placed in a culture flask at a concentration of 50 eggs/μl. The eggs were incubated at 27°C in the dark until third stage larvae were present and were subsequently used for infection trials or to extract infective stage-three larvae (L3).

Fresh L3 were obtained from *Ascaris* eggs using the method described by Urban et al. [25]. In brief, *Ascaris* eggs, cultured *in vitro* were treated for 1 hr with commercial bleach and subsequently washed 3 times with phosphate buffered saline (PBS) of 37°C after which the eggs were transferred into an Erlenmeyer flask containing glass beads and a magnetic stir bar and stirred very slowly (60 rpm) to induce hatching. After 15 min the suspension was poured onto a layer of cotton wool placed on top a Baermann apparatus with PBS at 37°C and left overnight. The next day, the larvae in the neck of the funnel were collected and washed 3 times in PBS. L3 extracts were prepared by grinding the collected larvae with a mortar and pestle that was placed in a bath of liquid nitrogen. The larval homogenate was transferred to a 15 ml tube and mixed with PBS and proteinase inhibitor cocktail (1:100) (Sigma, Diegem, Belgium). The homogenate was then inverted at 4°C for 2 hrs followed by centrifugation for 30 min at 10,000g at 4°C. The supernatant (L3 PBS) was removed and kept on ice. The pellet was resuspended in PBS with 0.05% Tween-20 solution and fresh proteinase inhibitor cocktail (1:100) was added. The mixture was inverted at 4°C for 2 hrs and the supernatant (L3 PBST) was removed after centrifugation and stored on ice. Finally, the remaining pellet was resuspended in PBS containing Triton X-100 (2%) and proteinase inhibitor cocktail (1:100) and subsequently inverted at 4°C for 2 hrs followed by centrifugation. The supernatant (L3 Triton) was collected and stored on ice. Subsequently, all extracts were sterilised by filtration (0.22μm) and the filtrate concentrated at 4°C using a Centriprep centrifugal filter with YM-3 membranes (Millipore, Overijse, Belgium). Protein concentration was determined by the BCA method (Pierce, Rockford, USA) and the extracts were stored at -80°C until use.

In order to obtain L3 excretory-secretory (E/S) products, freshly obtained *A*. *suum* L3 were incubated at 37°C and 5% CO_2_ at a concentration of 5,000 larvae/ml in DMEM with 4,5 g/L Glucose, L-Glutamine and Pyruvate (Thermo Fisher, Erembodegem, Belgium) containing 1% Penicillin-Streptomycin (P/S) (5,000 u/ml Penicillin and 5,000 μg/ml Streptomycin, Thermo Fisher), 1% Kanamycin (10000 μg/ml, Thermo Fisher), 1% Amphotericin B (250 μg/ml, Sigma) and 0,5% Gentamicin (10 mg/ml, Thermo Fisher). The culture fluid was collected daily and filtered using 0.2 μm membrane disc filters (Supor 200, Zaventem, Belgium), concentrated and dialysed against PBS in an Ultrafiltration Stirred Cell (Millipore) using a 10kDa cut-off filter membrane (Millipore). The L3 E/S material was then stored in aliquots at -80°C.

In order to obtain lung L3 and intestinal L4 and L5 stages, piglets were infected with approximately 100,000 infective *A*. *suum* eggs and sacrificed 7, 14 and 28 days post-infection, respectively. The lung L3 and intestinal L4 were collected from minced lung tissue or intestinal content that was placed on a modified Baermann device as described by Slotved et al. [26]. L5 larvae were collected from the intestinal content by hand. Larvae were washed excessively using PBS 4°C and stored at -80°C. Adult worms were collected from the intestines of infected pigs from commercial farms that were being processed as part of the normal work at a local abattoir in Ghent, Belgium. Protein extracts from all life stages were produced as described above for the L3 stage. Finally, extracts from *A*. *lumbricoides* L3, were obtained after sonication as previously described by Vlaminck et al., [27].

### Inducing pre-hepatic immunity in pigs by experimental trickle infection

Eight pigs were divided into 2 groups of 4 pigs. Pigs of group B were trickle infected 5 times a week with approximately 100 infective *A*. *suum* eggs in the feed for a period of 30 weeks. Pigs of group A were used as challenge controls. Two other pigs were euthanized before the start of the infection trial and used as negative controls. After the 30-week infection period, all pigs of group A and B were treated with a single dose of 5mg/kg fenbendazole (MSD, Brussels, Belgium). One week after treatment, all pigs were infected with 2,000 infective *A*. *suum* eggs. All pigs were euthanized 14 days post challenge infection. During necropsy, the number of white spots, characteristic lesions on the liver caused by migrating *A*. *suum* larvae, was recorded and mesenteric lymph nodes from the small intestine, caecum and colon were collected and immediately processed as described below. Pieces of small intestine of approximately 1 meter were flushed three times with 50ml of PBS to rinse out all larvae in the intestinal lumen. The rinsing solution was collected and passed over a 200 μm mesh sieve. The remaining debris on top of the sieve was collected and examined for intestinal L4 larvae. After rinsing, the pieces of small intestine and the caecum and colon were cut open longitudinally and washed gently with excessive volumes of lukewarm tap water to remove any remaining intestinal content. Subsequently, mucus was collected by gently scraping the luminal side of the pieces of intestine with a microscope slide. During the experiment, blood samples for serum were collected from all pigs every 2 weeks.

### Isolation and *in vitro* cultivation of mononuclear cells from local intestinal lymph nodes

Single-cell suspensions of mesenteric lymph node cells (LNC) were prepared by mechanical disaggregation through a sterile stainless steel gauze. The LNC in ice cold PBS were centrifuged at 150 g for 10 min at 4°C, the supernatant removed and LNC resuspended in DMEM growth medium supplemented with 4,5 g/L Glucose, L-Glutamine and Pyruvate (Thermo Fisher) containing 1% P/S (Thermo Fisher). The mononuclear cells (monocytes and lymphocytes (MNC)) were separated by the addition of LymphoPrep and centrifugation at 800g (without brake) for 30 min at 4°C. The MNC were collected from the interphase and washed twice with DMEM + 1% P/S + 2% heat-inactivated foetal bovine serum (Moregate, Australia & New Zealand) and the cells were subsequently suspended in DMEM_complete_. (DMEM + 1% P/S + 1% Non-Essential Amino Acids (Thermo Fisher), 1% Kanamycin (10 mg/ml, Thermo Fisher), 1% Amphotericin B (250 μg/ml, Sigma) and 0.1% of a 0.35% β-mercaptoethanol solution). Viable MNC were counted, the concentration adjusted to 5.0 10^6^ cells/ml in DMEM_complete_ and cultured for 4 days in tissue culture flasks at 38°C and 5% C0_2_. Finally, the culture supernatant (SN) was collected, filtered using 0.2 μm membrane disc filters (Supor 200), concentrated using Centriprep centrifugal filter units with YM-3 membranes (Millipore) and stored in aliquots at -80°C.

### Purification of antibodies from mucus

An equal volume of ice-cold PBS was added to the mucus collected from small intestine, caecum and colon and subsequently homogenised using an Ultrathurax mixer (2 min at 15,000 rpm). This mixture was then centrifuged for 15 min at 10,000g at 4°C, the supernatant was collected and centrifuged again as before. After this second centrifugation step, the supernatant was stored at -80°C. At a later time, antibodies were purified from this supernatant using Protein-A agarose beads (Sigma) following the manufacturer’s protocol. Purified mucosal antibodies were stored at -20°C until used.

### SDS-PAGE, Western blotting and staining procedures

Protein extracts (5 μg) were mixed with 5x sample buffer (60 mM Tris-Cl pH6.8, 2% SDS, 10% glycerol, 5% β-mercaptoethanol, 0.01% bromophenol blue) and put into a boiling water bath for 5 min. Afterwards, the samples were applied to 15% SDS-PAGE gels and separated by electrophoresis in Tris-Glycine buffer (Tris 250mM, Glycine 200mM, SDS 1% w/v). Protein bands were visualized using SimplyBlue Safestain (Thermo Fisher) or SilverStain kit (Thermo Fisher). Glycoproteins were stained by the use of the ProQ Emerald 300 gel stain kit (Thermo Fisher).

For Western blotting, SDS-PAGE gels were blot transferred to PVDF membranes (Millipore) or nitrocellulose membranes (Thermo Fisher) and blocked in PBS + 0.2% Tween80 (PBSt80) or in PBS + 0.2% Tween20 (PBSt20) + 5% Blotting-Grade Blocker (BioRad). The blots were probed for 2 hrs (1ml/lane PBSt80 containing approximately 5 μg/ml antibodies purified from mucus or the concentrated culture supernatant of MNCs). The following conjugates and dilutions were used: goat anti-pig IgG-HRP conjugated (Sigma) (1/10,000), goat anti-pig IgA-HRP (Abcam, Cambridge, UK) (1/5,000). The immunoreactive antigens were visualised by chemiluminescent substrate (5ml 0.1M Tris pH 8.6 + 11μl 90mM p-coumaric acid (Sigma) + 25μl 250mM luminol (Sigma) + 15μl of 10% (v/v) H_2_O_2_).

### Purification of the L3 total lipids

L3 PBS extract was subjected to a Folch method [28] for the extraction of total lipids. In short, L3 PBS extract was mixed with chloroform/methanol (2/1) to a final volume 20 times the volume of the original extract and incubated for 20 min. The homogenate was centrifuged and the liquid phase recovered and washed with 0.2 volume of 0.9% NaCl solution. The mixture was centrifuged at 2,000 rpm to separate the two phases. Both upper and lower phases were collected separately and evaporated at 90°C under a fume hood. The dried lipid pellets were stored at -80°C until further use.

### Chemical and enzymatic degradation of the L3 PBS extract and As12

To investigate the composition of the As12 antigen, total L3 PBS extract was incubated with different enzymes and submitted to different chemical treatments. For the enzymatic degradation, the L3 PBS extract was treated overnight at 37°C with pronase, lipase and trypsin at pH 8.0 and pepsin at pH 3.0 (all from Sigma). Additionally, L3 PBS extract was treated overnight at 37°C or 60°C in 20mM periodic acid, 1M NaOH, 1M Trifluoracetic acid and 1M HCl or in PBS at 37°C, 60°C or 90°C. Purified As12 was treated in 48% aqueous HF at 4°C for two days, then lyophilized and used for Western blotting. The presence of phosphorylcholine (PC) on the antigen was confirmed by screening with TEPC-15 monoclonal IgA antibodies (Sigma) on western blot. The deglycosylation of The L3 PBS extract with PNGase F was performed under denaturing conditions according to the manufacturers’s protocol (Promega). RNAse B was used for treatment control. The As12 antigen was also incubated in 50mM NaPO_4_, pH 5.0 for 2 days at 37°C with 0.1 unit ß-N-acetylglucosaminidase from jack bean (Sigma). After incubation, samples were boiled in 5x sample buffer, run on SDS-page, blotted onto PVDF membranes (Millipore) and recognition of the As12 antigen checked by incubation with mucosal antibodies from pigs of group B with 5μg/ml of antibodies/lane.

### Glycan analysis

For glycan analysis, aliquots of As12 were treated with PNGase-F, -A and chemical β-elimination to release N- and O-glycans, respectively. Released glycans were analysed by MALDI-TOF-MS after derivatisation with 2-aminobenzoic acid or permethylation, as described previously [29, 30].

### Monosaccharide composition analysis

Approximately 2 μg As12 was hydrolysed in a glass vial with 50 μl 4 M TFA at 100°C for 4 hrs, dried under nitrogen, then monosaccharides labelled with 10 μl 2-aminobenzoic acid (2-AA) labelling mix (48 mg/ml 2-AA, 1 M 2-picoline-borane dissolved in 30% acetic acid / DMSO) followed by 2 hrs incubation at 65°C. For HPLC, 10 μl labelled sugars was added to 90 μl 0.6% sodium acetate, and 25 μl applied to a Superspher 100 RP-18 column 250 x 4 mm (Merck). Buffers were: A = 0.1% butylamine, 0.5% phosphoric acid, 1% tetrahydrofuran; B = 0.05% butylamine, 0.25% phosphoric acid, 0.5% tetrahydrofuran, 50% acetonitrile. Run conditions were 0.5 ml/min, starting at 8% buffer B for 5 min, 8–25% buffer B over 25 min, 25–100% B over 2 min, maintained for 10 min. Monosaccharide standards (500 pmol Glc, Gal, Man, Fuc, Xyl, GlcNAc, GalNAc) were treated with TFA, labelled, and ran as above.

### Antibody labelling of larvae

A total of 40,000 L3 were incubated for 1 hr at 37°C in 1ml RPMI (Thermo Fisher) with 20μg purified mucosal antibodies from pigs from group A, group B and from negative controls or with TEPC-15 antibodies (Sigma) at a concentration of 1/500. After the incubation, larvae were washed three times with 1ml RPMI 37°C before being resuspended for 30 min in 1ml RPMI at 37°C containing FITC labelled anti-pig IgG (Bethyl laboratories, Montgomery, TX, USA) at a concentration of 1/1,000 or FITC labelled anti Mouse IgA at a concentration of 1/2,000 (Thermo Fisher). All incubations with FITC-conjugated antibodies were conducted in the dark. Finally, the larvae were washed four times with 1ml of PBS 37°C to remove any remaining secondary antibodies and put on a glass slide for fluorescent microscopy analysis. All labelling experiments were performed in triplicate.

To determine whether the stained L3 actively shed the As12 antigen, larvae were stained with 20μg of purified mucosal antibodies from pigs from group B as described above. After staining and washing, half of the larvae were killed by freezing them for 10 min at -80°C. The other half was kept in RPMI at 37°C. After this, the number of stained larvae in 3 aliquots of both groups was counted by fluorescence microscopy. This assessment was repeated after 1, 2, 3, 4 and 24 hrs.

### Vaccination experiment in piglets

Twelve piglets (approximately 10 weeks old) were divided into two groups of 6 pigs. Each pig in Group A was vaccinated with total lipid fraction purified from L3 PBS extract that was obtained from 200,000 L3 and dissolved in 0.5 ml sterile PBS + 0.5 ml Alhydrogel (AlOH). The control pigs of group B were injected with 0.5 ml sterile PBS + 0.5 ml AlOH. Pigs were immunized three times by intramuscular injection at day 0, 14 and 28 of the experiment. One week after the final immunization, all pigs were experimentally infected with 1,000 infective *A*. *suum* eggs in 5ml of tap water by oral intubation. Two weeks after infection, at day 49 of the experiment, all pigs were euthanized and L4 larvae were recovered from the small intestine as described above and counted. Blood samples were taken at the start of the trial, one week after the third immunization and at the time of necropsy to evaluate seroconversion against the As12 antigen following vaccination.

### ELISA

Indirect ELISA determined antibody recognition of the As12 antigen by pig sera or human plasma samples. ELISA plates were coated with antigen overnight in carbonate buffer (pH 9.6) at 4°C. Plates were coated with 1μl/ml of total lipid extract purified from 200,000 L3 which was dissolved in 50μl UPW. After three washes with PBSt, the plates were blocked with 100μl/well blocking buffer (5% milk powder (w/v) or 5% heat treated fetal calf serum in PBS) for 2hrs at 4°C. Sera or plasma samples were added in duplicate at a dilution of 1/250 in PBSt for 2hrs at 4°C. The plates were washed again as before and incubated with the conjugate (goat anti-pig IgG-HRP (Sigma) (1/10,000), goat anti-human IgG4-HRP (Southern biotech) (1/2,000)) in blocking buffer. Plates were incubated for 1hr at 37°C. O-phenylenediamine 0.1% in citrate buffer (pH 5.0) served as substrate and after a 10 min incubation period in the dark, the development reaction was stopped by adding 50μl of 4M H_2_SO_4_ to all wells and optical density (OD) was measured at 490 nm.

Sera from the trickle infected pigs described above were used to evaluate antibody response over time. Additional pig sera were obtained from an experimental infection trial performed by Nejsum et al., [31] where piglets 10 weeks of age were infected twice a week in the feed for a total of 14 weeks with *A*. *suum* and *Trichuris suis* (25 and 5 embryonated eggs kg−1 day−1, respectively). Serum and faecal samples were collected at the start of the trial (W0) and 7 (W7) and 14 (W14) weeks after the first infection. Pigs were euthanized 14 weeks after the start of the experiment and the number of macroscopic worms present in the small intestine counted. The negative control was a pooled serum sample from 4 10-week old piglets without previous exposure to *A*. *suum*. The positive control was a pooled serum sample from 4 pigs after 14 weeks of daily infection with 100 *A*. *suum* eggs [12]. Reactivity of pig sera to the antigen is shown in ODr (Optical Density ratio). (ODr _sample_ = (OD _sample_−OD _negative control_) / (OD _positive control_−OD _negative control_)).

Plasma samples from *A*. *lumbricoides* infected humans were collected as previously described [32] from individuals living in Mainang village on Alor Island (Province of East Nusa Tenggara, Timor, Indonesia), an area with high *Ascaris* prevalence (>30%). A subset of 25 plasma samples of which all individuals had *A*. *lumbricoides* eggs in their stool was evaluated for anti-As12 IgG4 antibodies. A total of 24 plasma samples from individuals with hookworm infection used in this study were from people living in the East Sepik region of Papua New Guinea where *A*. *lumbricoides* was not present. Non-endemic control sera were from American subjects in St. Louis, MO, USA. Reactivity of human sera to the As12 antigen is shown in OD.

### Binding of anti-PC antibodies to As12

TEPC-15 antibodies (Sigma) or pooled mucosal antibodies of pigs from group B of the trickle infection experiment were dissolved in PBSt at a dilution of 1/250 or 10μg/ml respectively and subsequently pre-incubated with PC-Cl salt (Sigma) at different concentrations (0, 10, 25 μg/ml final concentration) for 30 min at room temperature. Following pre-incubation, the antibody mixtures were added to an As12-coated ELISA plate for 2hrs at 4°C. After washing the plates three times with PBSt, the TEPC-15 antibodies were detected by HRP labelled anti Mouse IgA at a concentration of 1/2,000 (Bethyl laboratories) and the mucosal antibodies were detected by HRP conjugated goat anti-pig IgG (Sigma) at a 1/5,000 dilution in blocking buffer for 1hr at 37°C. Finally, the plates were washed, developed and read as described above.

### Statistical analysis

All statistical analyses were performed using Graphpad Prism 6.0e for MacOSx. The Mann-Whitney U-test for pairwise comparison was used to compare the group means of the immune pigs and the challenge controls during the vaccination trial or to compare anti As12 antibody levels between different groups of human or pig plasma or serum samples. T-tests were performed to test for differences in the percentage of stained larvae or antibody reactivity of the two different experimental groups during the vaccination experiment. A paired t-test was used to detect the first time point at which infected pigs showed a significant higher ELISA reactivity to As12 antigen compared to the start of infection. Possible correlations between antibody responses and parasitological data (EPG or worm counts) were assessed using the Spearman's rank correlation coefficient. The use of computed Receiver Operating Characteristic (ROC) curves allowed for the determination of test sensitivity and specificity and to select an appropriate cut-off. Probability (P) values < 0.05 were considered to indicate significant differences.

### Ethical clearance

All animal experiments were conducted in accordance with the E.U. Animal Welfare Directives and VICH Guidelines for Good Clinical Practice. Ethical approval to conduct the studies was obtained from the Ethical Committee of the Faculty of Veterinary Medicine, Ghent University. The collection of adult *A*. *lumbricoides* worms was performed during a trial performed by Mekonnen et al., in 2013 [24]. This study was approved by the ethical committee of Jimma University, Ethiopia, Ghent University and Antwerp University, Belgium. The Ethical Committee of the University of Indonesia, Jakarta approved the Alor Island study as previously described [32]. The Human Investigations Institutional Review Boards of Case Western Reserve University and the Papua New Guinea Medical Research Advisory Committee approved all protocols. The Institutional Review Board at Washington University School of Medicine in St. Louis, MO, USA approved our use of anonymized patient samples for the development of serological tests for helminth infections. Since written consent is not consistent with cultural norms on Alor Island, oral informed consent was obtained from all adults or, in the case of children, from their parents. The participant’s oral consent was noted on a survey questionnaire. The ethical board of the University of Indonesia and the institutional review boards in Germany and the USA approved the use of oral consent.

## Results

### Immunization of pigs by trickle infections

Immunity was induced in 4 pigs (Group B) trough trickle infection with 100 *A*. *suum* eggs 5 times a week for a period of 30 weeks. After a subsequent challenge infection with 2,000 infective *A*. *suum* eggs, the number of larvae and liver white spots were significantly reduced in these pigs when compared to control pigs who were not trickle infected (Group A) (Table 1). Challenge control pigs from Group A showed an average of 72.5 ± 43.3 liver white spots and 105 ± 81.3 L4’s in the intestine. In contrast, immunized pigs showed a 99% reduction in number of white spots on the liver (2.0 ± 1.8) and a 100% reduction in the number of L4 stage larvae in the intestine 2 weeks post-challenge, indicating the presence of intestinal immunity against infectious *A*. *suum* L3 in these pigs.

**Table 1:** Long term trickle infection of pigs with *A*. *suum* results in reduced liver white spots and L4 recovered from the small intestine 14 days post challenge infection with 2,000 infective eggs.

Group	n	Immunization protocol (30 weeks)	White spots	L4
A	4	No infection	72.5 ± 43.3	105.0 ± 81.3
B	4	100 eggs/day, 5 days/week	2.0 ± 1.8*	0.0 ± 0.0*

* Significant difference when compared to control group (Group A) (P<0.01).

### Local antibody responses of immune pigs to L3 extracts

**Fig 1:**
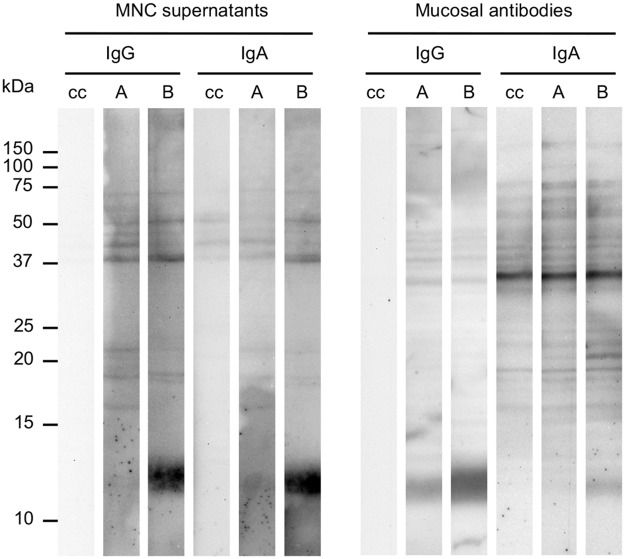
The detection of a 12kDa *A*. *suum* L3 antigen in the L3 PBS extract by intestinal antibodies. IgG and IgA antibodies purified from the supernatant of purified mononuclear cells (MNC) from mesenteric lymph nodes or the intestinal mucus were used to screen the L3 PBS extract on Western blot. Antibodies from immune pigs (Group B) strongly react to an antigen migrating at 12 kDa. Pigs from the challenged control group (Group A) also had antibodies against the 12kDA antigen, but the reactivity on Western blot was lower than in immune pigs. Conjugate alone (cc) did not react with the antigen.

Antibodies from the intestinal mucus and from MNC culture supernatant were used to detect immunogenic antigens in *A*. *suum* L3 protein extracts (Fig 1). Several antigens were recognized by IgG and IgA antibodies isolated from both the challenge control pigs and the trickle infected pigs. One zone of approximately 12kDa in size was recognised by IgG and IgA antibodies from the immunized pigs (Group B). Although this zone was also detected by the challenge control animals (Group A), recognition was generally much more intense in the immune animals (Group B). This antigen, from here on out referred to as As12, was also detected in the water-insoluble protein fractions of L3 (S1 Fig). In addition, there was also increased recognition of a 21kDa antigen in the L3 PBS extract by mucosal IgA antibodies of immunized pigs.

### Presence of As12 in different life stages of *A*. *suum* and *A*. *lumbricoides*

**Fig 2:**
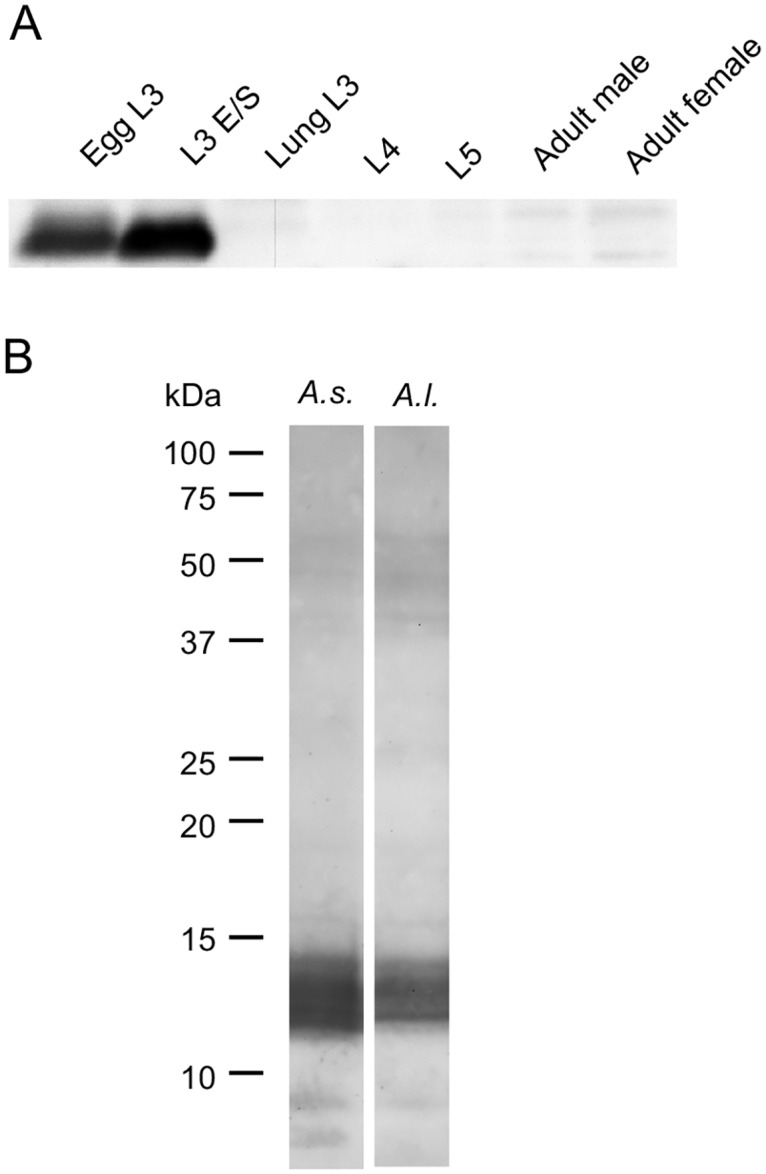
The As12 antigen is produced and secreted by the early L3 stage of *A*. *suum* and is also present in *A*. *lumbricoides* L3. (A) The detection of the As12 antigen by IgG antibodies purified from the intestinal mucus of *A*. *suum* immune pigs in PBS extracts of different *A*. *suum* life stages (Egg L3, L3 E/S, Lung L3, L4, L5, Adult male and female worms) and (B) in the PBS extract of *A*. *suum* and *A*. *lumbricoides* L3.

An immunoblot of the PBS extracts of the different life stages of *A*. *suum* showed that the recognition of the As12 antigen was restricted to the early L3 stage and its E/S products (Fig 2A). The antigen was absent from the lung stage larvae onwards. In the L3 PBS extract from *A*. *lumbricoides*, a band of the same molecular weight as As12 was recognised (Fig 2B).

### Properties of As12

**Fig 3:**
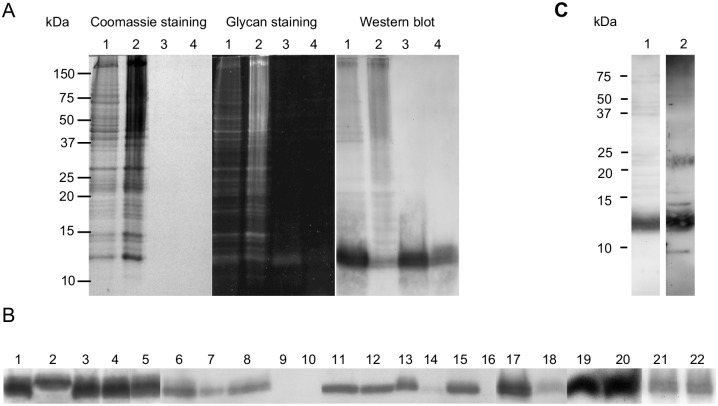
Exploring the properties and composition of the As12 antigen. (A) Coomassie staining, carbohydrate staining and Western blot developed with mucosal IgG antibodies from immune pigs (Group B) of the complete L3PBS extract (1), the pellet after Folch extraction (2), the upper ‘methanol phase’ (3) and the lower ‘chloroform phase’ (4). (B) Recognition of the As12 antigen by mucosal IgG antibodies from immune pigs after chemical or enzymatic treatments of the *A*. *suum* L3 PBS extract: untreated (1&17); overnight digestion at 37°C with pronase (2); lipase (3); trypsin (4); pepsin (5); overnight incubation at 37°C (6), 60°C (7) and 90°C (8); overnight incubation with 20mM periodic acid at 37°C (9) and 60°C (10), 1M NaOH at 37°C (11) and 60°C (12), 1M trifluoracetic acid at 37°C (13) and 60°C (14); 1M HCl at 37°C (15) or 60°C (16); 48 hrs incubation in 48% HF at 4°C (18); overnight incubation at 37°C without (19) or with PNGase-F (20) and 48 hrs. incubation at 37°C with (21) or without (22) Jack Bean ß-N-Acetylglucosaminidase. (C) Recognition of *A*. *suum* L3 PBS antigens by purified mucosal IgG antibodies from immune pigs (1) and specific anti-phosphorylcholine antibodies (TEPC-15) (2).

The As12 antigen could be visualised by a staining for carbohydrates or after immunoblotting but not by conventional protein staining methods like silver staining or coomassie staining (Fig 3A). The As12 antigen could be purified from the L3PBS protein extract by performing a Folch extraction for the isolation of total lipid. The antigen appeared to be the sole recognized antigen in both the upper methanol and lower chloroform phase after Folch extraction (Fig 3A).

To further explore the specific properties and composition of the As12 antigen, the antigen was subjected to different chemical and enzymatic treatments. The immunoblot reaction to the As12 antigen by purified mucosal antibodies following exposure of the L3 PBS extract to a range of chemical and enzymatic treatments is shown in Fig 3B. Overnight treatment of the L3PBS extract with pronase, lipase, trypsin or pepsin did not affect antibody recognition of the As12 antigen. Similarly, heating the L3PBS extract to 60°C or 90°C overnight did not reduce the recognition of As12, as well as treatment with 1M NaOH. Incubating the L3 PBS antigen with PNGaseF to cut off N-linked glycan structures or subjecting it to ß-N-acetylglucosaminidase treatment for the liberation of terminal ß-linked N-acetylglucosamine and N-acetylgalactosamine residues also did not diminish the recognition of the As12 antigen. The only chemical reactions that reduced or prevented recognition of As12 by porcine antibodies were treatment with 20mM periodic acid at 37°C or 60°C or 1M trifluoracetic acid and 1M HCl at 60°C. Finally, treatment of the As12 fraction with 48% HF for 48 hrs at 4°C for the removal of any PC groups on the antigen also reduced the immune recognition. The presence of a PC group on the As12 antigen was further confirmed by Western blot using specific anti PC antibodies (TEPC-15) (Fig 3C). Next to the As12 antigen, several other PC-bearing antigens were detected in the *A*. *suum* L3 extract.

Mass spectrometric analysis of glycans released from As12 after enzymatical treatments with PNGase-F and -A or by chemical β-elimination provided no indications for the occurrence of common N- or O-glycans on As12. Monosaccharide composition analysis indicated that the glycan portion of As12 consists mainly of GalNAc and GlcNAc in an approximate 1:2 ratio, with a minor amount of Glc (S2 Fig).

### Immunolocalization

**Fig 4:**
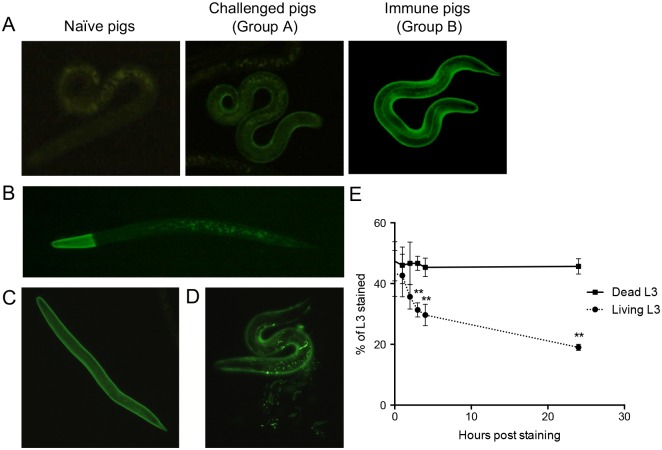
Mucosal antibodies from immune pigs recognize antigens on the surface of *A*. *suum* L3 that likely contain phosphorylcholine groups and are actively secreted by living larvae. (A) UV-microscopy images (400x) of *A*. *suum* L3 larvae stained by mucosal antibodies from naïve pigs, challenge control pigs (Group A) or immunized pigs (Group B) and incubated with FITC labeled anti-pig IgG. (B) UV-microscopy image (400x) of *A*. *suum* L3 larva partially stained by mucosal antibodies from immune pigs and incubated with FITC labeled anti-pig IgG. (C) A UV-microscopy image (400x) of an *A*. *suum* L3 larva stained by anti-PC antibodies and (D) a stained larva in the process of shedding these antigen-antibody complexes. (E) The percentage of *A*. *suum* L3 stained by mucosal antibodies from immune pigs decreased over time when L3 were kept alive in culture media (dotted line) but not when L3 were killed after staining (full line) (P < 0.01).

Freshly hatched *A*. *suum* L3 were incubated with antibodies purified from the intestinal mucus of immunized pigs (Group B). On Western blot these antibodies mainly recognized the As12 antigen (Fig 3C). Antibodies bound to the surface were detected with FITC labelled anti-pig total IgG. This showed complete staining of the outer surface of larvae that had shed their L2 sheath (Fig 4A). Other larvae with partially shed L2 sheaths showed only incomplete staining (Fig 4B). To further verify that the reactivity with mucosal antibodies was directed against the As12 antigen, L3 larvae were also incubated with monoclonal antibodies directed against PC. This labelling also resulted in staining of the L3 surface (Fig 4C). Live L3 appear to shed off the antigen-antibody complexes (Fig 4D). As a consequence, the number of L3 stained by mucosal antibodies diminished significantly over time when stained larvae were kept alive in culture. In contrast, when larvae were killed after staining, the number of stained L3 did not diminish over time (Fig 4E).

### Evaluation of As12 as a vaccine antigen

**Fig 5:**
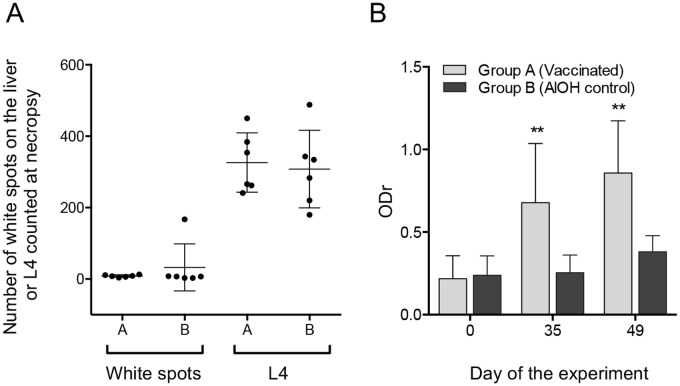
Vaccinating pigs with a purified As12 fraction in combination with AlOH adjuvant does not induce protection against subsequent challenge infection despite increased serum antibody responses to the antigen. (A) The number of white spots counted on the liver and the number L4 recovered from the small intestine at necropsy 14 days after a challenge infection with 1,000 infective *A*. *suum* eggs in 6 pigs vaccinated with As12 + AlOH (Group A) and 6 adjuvant control pigs (GroupB). (B) The total IgG response in the serum directed against the As12 antigen fraction was significantly elevated in the vaccinated group one week after the third immunization, at the time of infection (Day 35) and the day of necropsy (Day 49) in comparison to the adjuvant control group (P < 0.01).

Results of the vaccination trial with the As12 antigen are shown in Fig 5A. Immunizing pigs with the total purified lipid fraction from *A*. *suum* L3, containing the As12 antigen, did not seem to induce protection to subsequent homologue challenge infection. There was no significant difference in number of liver white spots or number of L4 recovered from the intestine 14 days after a challenge infection with 1,000 infective *A*. *suum* eggs. Pigs of the vaccinated group did however show a significantly stronger IgG response against the As12 antigen compared to control pigs (Fig 5B).

### Evaluation of As12 as a diagnostic antigen for ascariasis in pigs and humans

**Fig 6:**
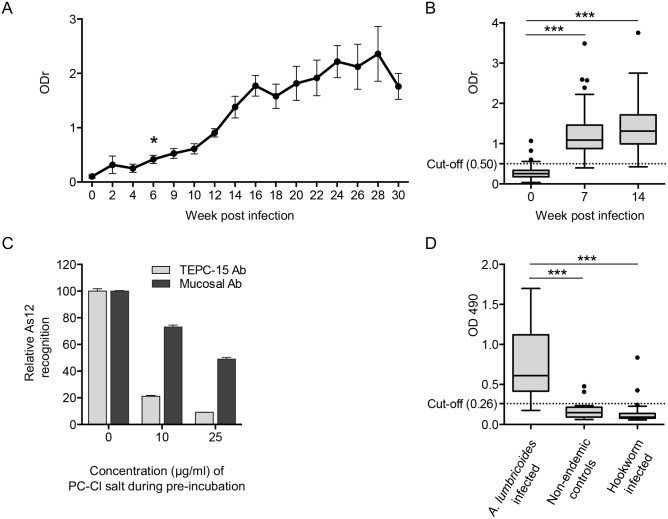
Pigs and humans infected with *Ascaris* develop antibodies to the As12 antigen. (A) The total IgG response to the As12 purified fraction over time in pigs exposed to trickle infections (500 *A*. *suum* eggs/week) over a 30-week time period shows significantly higher IgG responses from 6 weeks after the first challenge infection onwards (P < 0.05). (B) The serological response to the As12 fraction of 91 experimentally infected pigs increased significantly after 7 and 14 weeks of infection (P < 0.001). The optimal cut-off was determined by ROC analysis and set at 0.50 (dotted line). (C) Relative ELISA recognition of As12 by either TEPC-15 monoclonal antibodies or purified mucosal IgG antibodies from trickle infected pigs from group B after pre-incubation with increasing concentrations of PC-Cl salt (0, 10 and 25 μg/ml). (D) Total IgG response against As12 was significantly higher in the 25 *A*. *lumbricoides* infected humans than in the 20 uninfected or 24 hookworm-infected individuals (P < 0.001). The cut-off was set at an OD of 0.26 by ROC analysis (dotted line).

To evaluate whether the As12 antigen could serve as a serodiagnostic antigen to measure exposure to *Ascaris*, the serum IgG antibody responses against the As12 antigen in the immunized pigs of group A was measured over time (Fig 6A). Anti-As12 antibodies were significantly elevated from 6 weeks after initial infection. Subsequently, sera from 91 experimentally infected pigs (31) were analysed using the As12 ELISA and showed that the reactivity to the As12 antigen increased significantly in pigs that were continuously infected with *A*. *suum* eggs (Fig 6B). After ROC analysis, using the week 0 sera as *Ascaris*-negative and both week 7 and week 14 sera as *Ascaris* positive, the cut-off for positive individuals was placed at an ODr of 0.50. Using this cutoff, the sensitivity of the ELISA was 98.4% (95% CI: 95.3–99.7), the specificity was 95.5% (95% CI: 88.9–98.8%) and the area under the curve was 0.99 (± 0.049). No relationship was found between anti-As12 antibody levels and the faecal egg count after 7 or 14 weeks of trickle infection or the number of adult worms at necropsy.

We subsequently tested whether the antibody reactivity to As12 was solely directed against the presence of PC on the antigen. For this, both specific anti-PC monoclonal antibodies (TEPC-15) and pooled purified mucosal antibodies from trickle infected pigs were pre-incubated with increasing concentrations of PC-Cl salt to block the PC-binding sites. After pre-incubation, antibody preparations were used to detect As12 by ELISA. Pre-incubation of TEPC-15 antibodies with increasing concentrations of PC-Cl salt reduced binding reactivity to As12 in a concentration dependent way (Fig 6C). Although pre-incubation with PC-Cl salt also affected the binding reactivity of pooled purified mucosal antibodies, the relative level of inhibition was not as pronounced as for the TEPC-15 antibodies (79% vs 27% and 91% vs 51% reduction of recognition at 10 and 25μg/ml PC-Cl salt respectively). This suggests that the recognition of As12 by *Ascaris* infected pigs was not solely directed against PC.

Finally, the use of As12 as a serodiagnostic antigen was also validated with human samples. Twenty five human plasma samples from Indonesia from patients with proven *A*. *lumbricoides* infection (EPG >50) were analysed using the As12 ELISA (Fig 6D). In addition, 20 non-endemic plasma samples (USA) were used as negative controls and 24 plasma samples from individuals with hookworm infection were tested to evaluate possible cross reactivity. The levels of anti-As12 IgG4 in the human samples was significantly elevated in humans infected with *A*. *lumbricoides*. ROC analysis placed the optimal cut-off for the human As12 ELISA at an OD of 0.26. Using this cut-off, the sensitivity of the ELISA was 92.0% (95% CI: 74.0–99.0%), the specificity was 90.0% (95% CI: 68.3–98.8%) and the area under the curve was 0.96 (± 0.026). A total of 24 out of 25 *Ascaris* infected individuals (96%), 2 out of 20 non-endemic plasma samples (10%) and 2 out of 24 hookworm infected individuals (8.3%) were positive for anti-As12 IgG4 antibodies when this cut-off was employed.

## Discussion

This study describes the identification and characterisation of As12, a phosphorylcholine-containing glycolipid-like structure present on the surface of infective *Ascaris* L3 larvae. This antigen is targeted by the intestinal antibody response of pigs that have been previously exposed to infection. Both IgG and IgA isotype antibodies against As12 were produced by local antibody secreting cells and both Ig isotypes were detected in the mucus of infected pigs. Recognition of As12 seems to increase over time when pigs are continuously exposed to infection. The As12 antigen was also found in *A*. *lumbricoides* L3 and is being recognized by IgG4 from *A*. *lumbricoides* infected humans. As a result, an As12 based ELISA test could identify 98% of the *Ascaris* exposed pigs and 92% of *Ascaris* infected humans, whereas samples from non-endemic humans or hookworm infected individuals were negative except for two samples in each case.

An amorphous envelope called the surface coat covers the L3 cuticle surface and it presents the greatest interface between the parasite and its host. Our results suggest that the As12 antigen is likely to be a component thereof. The surface coat or “fuzzy coat” is not derived from the cuticle but from specialized secretory glands. It is rich in carbohydrates, is dynamically responsive to changing host environments or immune attack and can be rapidly shed upon binding by antibodies and/or immune cells [33–35]. This process of shedding seems to be actively regulated in *A*. *suum* L3 since no decrease in staining was seen in dead L3 stained with immune pig antibodies whereas the percentage of live stained L3 decreased significantly over time. Similarly, antibody recognition of surface antigens on *Toxocara canis* L3 or *Onchocerca cervicalis* microfilaria was also lost in a temperature dependent manner or after incubation with antimetabolites [36, 37]. This process of sloughing off antibody-bound antigens is likely to be one of the mechanisms used by *Ascaris* L3 in order to evade host immune responses. The high antigenicity of the As12 antigen, presented on the L3 cuticle surface, might engage the host immune system while the active shedding of the antigen after antibody binding prevents actual protective mechanisms to damage the mobile larvae. The antigen might also be actively secreted to influence the responses of nearby immune cells.

The 35 kDa carbohydrate antigen (CarLA) detected on L3 of the sheep parasite *Trichostrongylus colubriformis* [38] shows characteristics similar to As12 as it is also very resistant to multiple enzymatic and chemical treatments, except for those that targeted carbohydrate structures. Although no glycans were detected following PNGase-F and -A digestion or chemical β-elimination, monosaccharide analysis indicated a simple composition suggesting a more polysaccharide nature with repeating units composed of GalNAc and GlcNAc.

Just like As12, CarLA is solely produced and excreted by the infective L3 stage and not by any other life stage [39]. However, unlike As12, the CarLA antigen does not appear to possess any PC group [38]. This might explain why the As12 antigen could not be recognized by immune sheep sera or monoclonal antibodies directed against CarLA. The presence of a PC hapten is very common on nematode carbohydrates attached to a protein or lipid backbone. Previous studies have suggested that PC containing glycolipids from adult *Ascaris* worms have immunomodulatory properties [40–42]. It is possible that this As12 antigen has similar properties, but further research is required to confirm this.

Despite the apparent antibody response against As12 in vaccinated pigs, there was no protection against subsequent challenge infection. A recent study by Masure et al., [12] has shown strong eosinophil presence in the intestinal tissue of pigs which have developed a ‘pre-hepatic barrier’. Possibly, the vaccination failed to induce an effective local intestinal cellular response which appears necessary to kill and thereby prevent the worms from migrating. Furthermore, systemic vaccination might not have induced the production of anti-As12 antibodies at the level of the intestine, where immune-mediated killing of the larvae is expected to occur [12]. We recognize the unfortunate fact that the presence of anti-As12 antibodies in intestinal mucus was not evaluated in this experiment. It is likely that the synergetic cooperation between both antigen specific antibodies and cellular responses at the site of infection is key in stopping the migrating larvae.

The strong antibody responses that are mounted by the host against As12 may be useful for diagnostic purposes. Nearly all trickle-infected pigs showed strong development of anti-As12 antibodies, independent of whether they were harbouring adult worms in their gut or not. The onset of detectable antibodies was however first apparent after 6 weeks, which is similar to purified *Ascaris* haemoglobin antigen [15]. Infected humans also mounted a significant antibody response to the As12 antigen, whereas non-endemic or hookworm infected humans did not seem to recognize the antigen. This result, together with the fact that pre-incubation with PC-Cl salt of mucosal antibodies from trickle infected pigs did not completely prevent binding of As12 on ELISA suggests that recognition of As12 not only depends on the PC-group but possibly involves other parts of the antigen. More tests with sera from individuals with other well-characterized helminth infections need to be performed to further determine the species specificity of As12 as a serodiagnostic antigen.

In conclusion, in this study we used locally secreted antibodies from the intestine of pigs with a pre-hepatic immunity to identify one major immunodominant antigen in the extracts and E/S material of infective *Ascaris* L3. This As12 antigen is stage specific, of a glycolipid nature and is being actively secreted. Experimentally infected pigs or naturally infected humans develop a measurable antibody response against the As12, advocating its possible use as a diagnostic antigen. The exact structure of this antigen and its biological role during infection is yet unknown and deserves further clarification.

## Supporting Information

S1 Fig The detection of a 12kDa *A*. *suum* L3 antigen in the L3 Triton extract by intestinal antibodies. IgG and IgA antibodies purified from the supernatant of antibody secreting cells from mesenteric lymph nodes (ASC probes) or the intestinal mucus were used to screen the L3 Triton extract on Western blot. Antibodies from immune pigs (group B) strongly react to an antigen migrating at 12 kDa. Pigs from the challenged control group (Group A) also had antibodies against the 12kDA antigen, but the reactivity on Western blot was lower than in immune pigs. Conjugate alone (cc) did not react with the antigen.
(PDF)

S2 Fig HPLC analysis of AA-labelled monosaccharides obtained from the As12 fraction after TFA hydrolysis. The monosaccharide composition of the As12 fraction is identified by comparison with the equimolar monosaccharide standard mixture, indicating that the glycan fraction of the As12 fraction is composed of GalNAc, GlcNAc and a minor relative amount of Glc. Minor unknown peaks are indicated (*).
(PDF)

S1 Checklist STROBE Checklist. (DOC)
