# Supplementary material for: A Phosphorylcholine-Containing Glycolipid-like Antigen Present on the Surface of Infective Stage Larvae of Ascaris spp. Is a Major Antibody Target in Infected Pigs and Humans
Source: PLoS Negl Trop Dis. 2016 Dec 1;10(12):e0005166. doi: 10.1371/journal.pntd.0005166 (PMC5131908; doi:10.1371/journal.pntd.0005166)

Supplemental Figure 1: The detection of a 12kDa *A. suum* L3 antigen in the L3 Triton extract by intestinal antibodies. IgG and IgA antibodies purified from the supernatant of antibody secreting cells from mesenteric lymph nodes (ASC probes) or the intestinal mucus were used to screen the L3 Triton extract on Western blot. Antibodies from immune pigs (group B) strongly react to an antigen migrating at 12 kDa. Pigs from the challenged control group (Group A) also had antibodies against the 12kDA antigen, but the reactivity on Western blot was lower than in immune pigs. Conjugate alone (cc) did not react with the antigen.

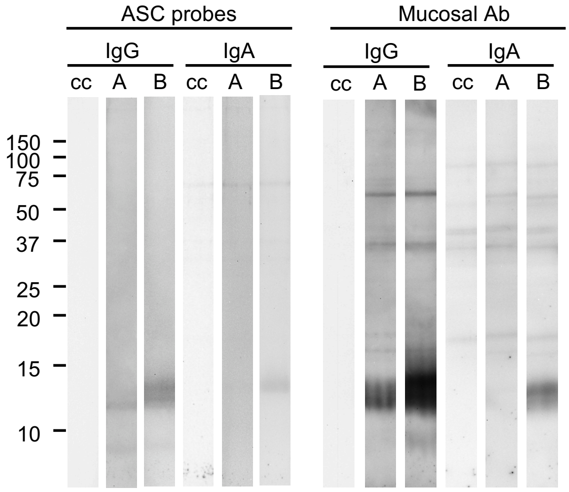

Supplement: S1 Fig — IgG and IgA antibodies purified from the supernatant of antibody secreting cells from mesenteric lymph nodes (ASC probes) or the intestinal mucus were used to screen the L3 Triton extract on Western blot. Antibodies from immune pigs (group B) strongly react to an antigen migrating at 12 kDa. Pigs from the challenged control group (Group A) also had antibodies against the 12kDA antigen, but the reactivity on Western blot was lower than in immune pigs. Conjugate alone (cc) did not react with the antigen. (PDF) [file pntd.0005166.s001.pdf]
