# Supplementary material for: A Phosphorylcholine-Containing Glycolipid-like Antigen Present on the Surface of Infective Stage Larvae of Ascaris spp. Is a Major Antibody Target in Infected Pigs and Humans
Source: PLoS Negl Trop Dis. 2016 Dec 1;10(12):e0005166. doi: 10.1371/journal.pntd.0005166 (PMC5131908; doi:10.1371/journal.pntd.0005166)

Supplemental Figure 2: HPLC analysis of AA-labelled monosaccharides obtained from As12 after TFA hydrolysis. The As12 monosaccharide composition is identified by comparison with the equimolar monosaccharide standard mixture, indicating that the glycan of As12 is composed of GalNAc, GlcNAc and a minor amount of Glc. Minor unknown peaks are indicated (*).

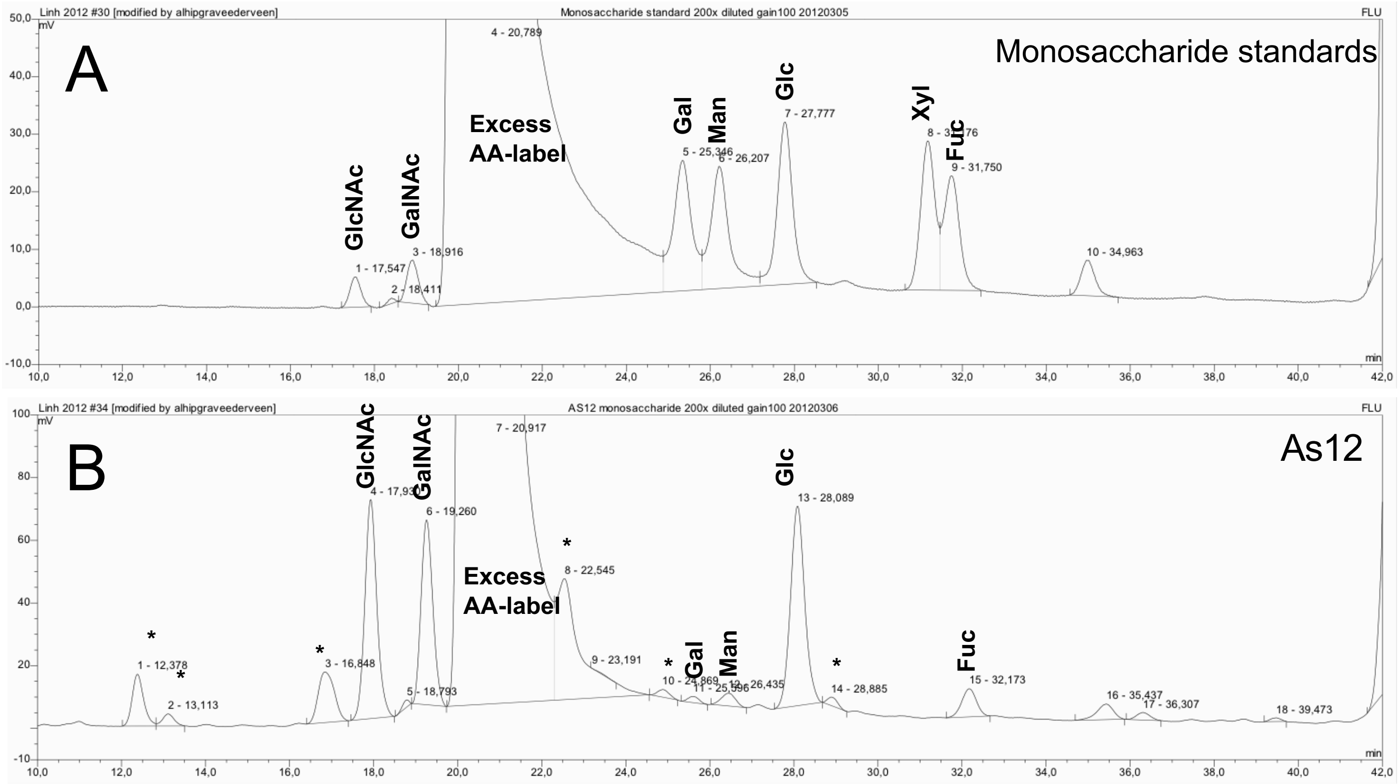

Supplement: S2 Fig — The monosaccharide composition of the As12 fraction is identified by comparison with the equimolar monosaccharide standard mixture, indicating that the glycan fraction of the As12 fraction is composed of GalNAc, GlcNAc and a minor relative amount of Glc. Minor unknown peaks are indicated (*). (PDF) [file pntd.0005166.s002.pdf]
